# Cephalometric analyses for cleft patients: a statistical approach to compare the variables of Delaire’s craniofacial analysis to Bergen analysis

**DOI:** 10.1007/s00784-021-04006-3

**Published:** 2021-05-29

**Authors:** Philine Henriette Doberschütz, Christian Schwahn, Karl-Friedrich Krey

**Affiliations:** 1 Department of Orthodontics, University Medicine Greifswald, Fleischmannstraße 42-44, 17475 Greifswald, Germany; 2 Department of Prosthetic Dentistry, Gerodontology and Biomaterials, University Medicine Greifswald, Fleischmannstraße 42-44, 17475 Greifswald, Germany

**Keywords:** Cephalometry, Cleft lip and palate, Delaire analysis, Correlation analysis, Factor analysis, Cluster analysis

## Abstract

**Objectives:**

Cephalometric analyses using lateral craniofacial radiographs are common diagnostic procedures for evaluating skeletal patterns. However, in patients with pronounced abnormalities like cleft lip and palate, standard cephalometric analyses and landmarks may not be suitable. This study aims to clarify whether the inclusion of landmarks less compromised by the cleft defect or located outside the cleft area results in a different cephalometric assessment of the viscerocranium. Delaire’s whole-skull analysis and Bergen analysis were examined for similarities and underlying common observations.

**Materials/methods:**

Based on the cephalometric evaluation of 217 patients with different types of non-syndromal cleft formation, Delaire and Bergen analysis were compared using three statistical methods: correlation analysis, factor analysis, and cluster analysis. Reproducibility was assessed by Bland–Altman plots, intraclass correlation coefficients, mean absolute differences, and coefficients of variability.

**Results:**

Although Delaire analysis and Bergen analysis are based on different concepts and landmarks, a majority of corresponding variables was found. Certain aspects of craniofacial base relation and craniospinal articulation are only assessed by Delaire analysis. All but one variable showed very good reproducibility.

**Conclusions:**

The inclusion of landmarks less compromised by or located outside the cleft area does not result in variables that provide a different assessment of the viscerocranial area.

**Clinical Relevance:**

The findings contradict the concept of invalidity of landmarks compromised by the cleft defect or located within the affected cleft area. Within the scope of its viscerocranial field of view, Bergen analysis appears to be on a par with Delaire analysis in the diagnosis of cleft patients.

**Supplementary Information:**

The online version contains supplementary material available at 10.1007/s00784-021-04006-3.

## Introduction

Cleft lip and cleft palate are multifaceted deformities affecting both the orofacial morphology and function. Their occurrence leads to marked differences in the dentofacial relation of patients compared to individuals without cleft formation [1]. To characterize the skeletal patterns of their patients, orthodontists and maxillofacial surgeons regularly conduct cephalometric analyses using lateral craniofacial radiographs. But for individuals with pronounced abnormalities — such as clefts — standard cephalometric analyses and normative values may not be suitable [2, 3]. It has not yet been conclusively clarified, how to adapt cephalometric analyses to complex situations in the midface region [3–8].

Considering the inherent morphological differences of cleft patients, the use of reference landmarks located outside the affected area has been proposed [9]. Jean Delaire and his team have considered a whole-skull analysis the best method to objectify and quantify deformities in cleft patients [10]. The architectural and structural craniofacial analysis by Delaire relies on individual proportions and aims to detect maxillofacial deformities and pathologic imbalances [11].

Although this analysis has been described as early as in 1979, it is still subject of research whether taking into account the whole skull offers diagnostic advantages over conventional cephalometric analyses of the viscerocranium. Current studies have focused on the applicability of the Delaire analysis compared with standard analyses as surgical decision tools in non-cleft patients [4–6].

We aim to clarify the suitability of the Delaire analysis compared with a conventional cephalometric analysis for assessing the viscerocranium in cleft patients.

## Materials and methods

The clinical sample consists of 217 patients of Western European descent with different types of non-syndromal cleft formation: unilateral cleft lip and palate (UCLP, n = 62), bilateral cleft lip and palate (BCLP, n = 78) and isolated cleft palate (CP, n = 77). This corresponds to all available, fully documented X-ray images from the archive of the former Wolfgang-Rosenthal Clinic Thallwitz (Germany) that met the following criteria: The patients had been treated according to the concept of late palate closure (lip closure during first six month of life and palatal operation in the fourth year of life). Patients had undergone dentofacial orthodontic and orthopedic therapy, but no orthognathic surgery had been performed before the cephalometric radiographs were taken. To ensure that most craniofacial growth had already occurred, only subjects with cervical vertebral maturation stage CS-5 or CS-6 were included in the study [12].

X-ray films (whole-skull X-ray, 4 m focus film distance, format 23.5 × 29.5 cm) were scanned into digital format (resolution: 300 dpi, gray shade: 16 bit, format: TIFF) using Intelli Scan 1600 (Quatographic Technology GmbH, Braunschweig, Germany) and transferred to dental imaging software Onyx Ceph (Image Instruments, Chemnitz, Germany). Radiographs scanned at a resolution of 300 dpi, as performed in this study, are comparable to analog cephalograms and sufficient for clinical purposes [13]. All tracings were performed by one investigator on a high-resolution monitor (Barco Nio MDNC-2123, Barco, Kortrijk, Belgium) in a darkened room.

The craniofacial morphologies of the patients were compared to the normative values from population means using the Bergen cephalometric analysis [14] and assessed for harmonic craniofacial relations using the whole-skull analysis by Delaire [11]. Since no linear measurements were made and only angles and distance ratios were calculated, the magnification factor of the X-ray recordings did not need to be considered. The Delaire analysis also describes the ideal positional relationship of spatial planes relative to anatomical landmarks. Therefore, in addition to the numerical analyses, a visual descriptive assessment of all subjects was made based on five categorical variables.

**Fig. 1:**
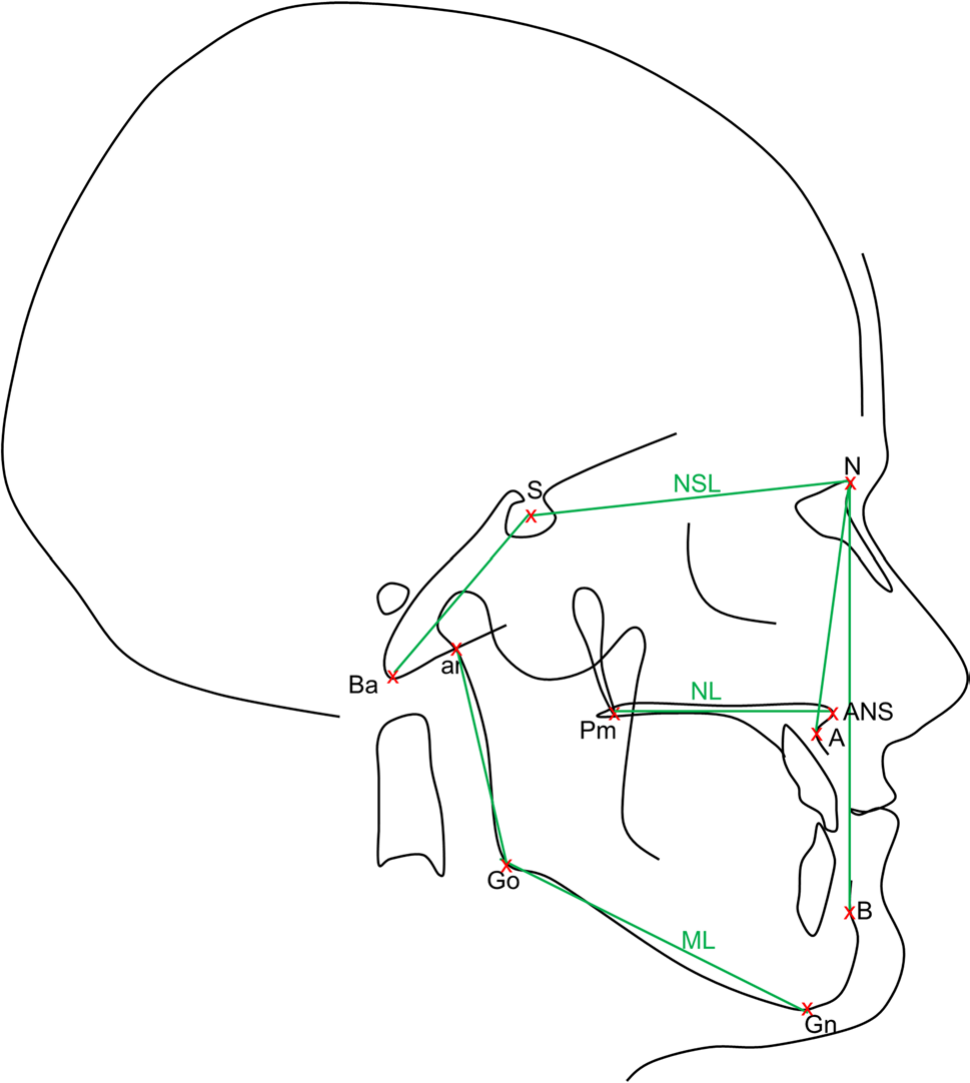
Visualization of the landmarks and reference lines used in the Bergen analysis. The positioning of landmarks and reference lines demonstrates that the Bergen analysis sets focus on the orofacial area

Table 1 and Fig. 1 summarize and visualize the Bergen analysis used in this investigation.

**Table 1:**
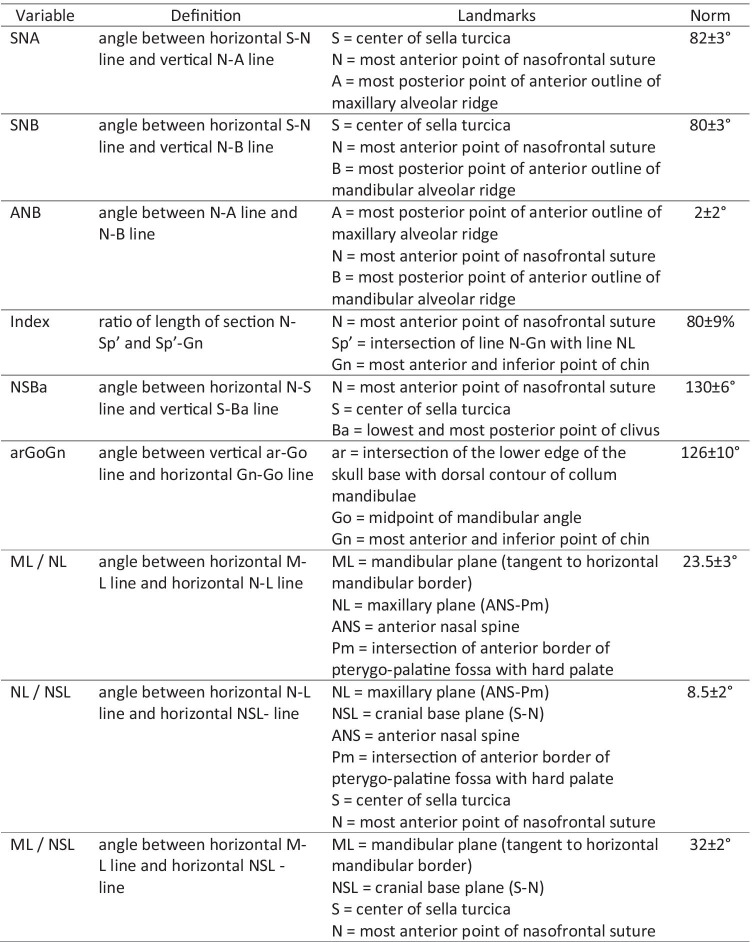
Variables, landmarks and normative values of the Bergen analysis

**Fig. 2:**
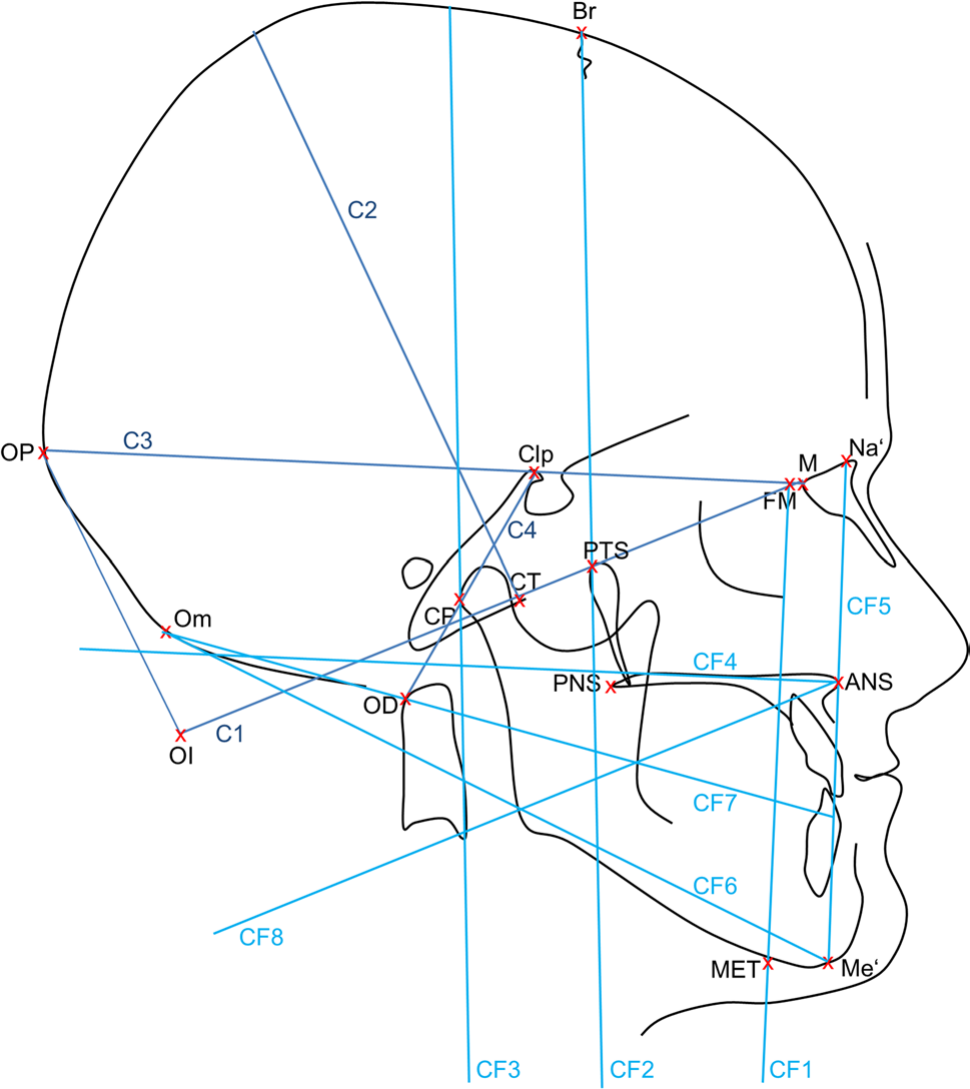
Visualization of the landmarks and reference lines used in the Delaire analysis. The positioning of landmarks and reference lines demonstrates that the Delaire analysis takes into account the entire cranial, facial and craniospinal area

Tables 2 and 3 and Fig. 2 summarize and visualize the Delaire analysis used in this investigation. Note that the nomenclature of the Delaire analysis was changed in a revised version [15]. The present study refers to the original nomenclature [11].

**Table 2:**
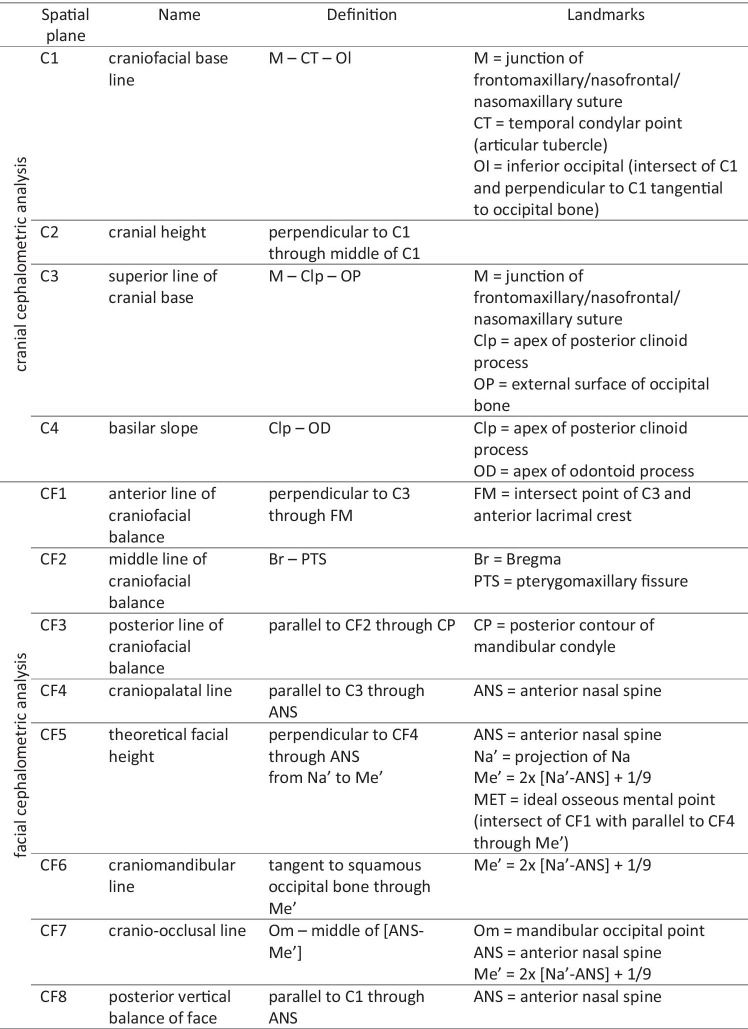
Reference lines and landmarks of the Delaire analysis

**Table 3:**
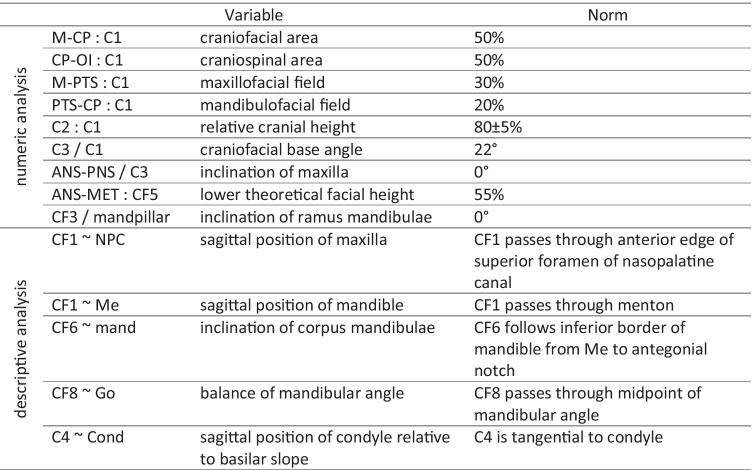
Variables and normative values of the Delaire analysis

To reduce model uncertainty, similarities and differences of the analyses by Delaire and Hasund (Bergen analysis) were studied in three ways [16]. First, Pearson correlations between continuous variables and Spearman correlations including the ordinal variables were graphed. Second, factor analysis was conducted to interpret the underlying structure rather than the variable level. The continuous variables were fitted by the maximum likelihood method [17]. The variable “Index” was excluded to avoid a Heywood case. The number of factors was chosen to get residuals less than 0.1, which resulted in eight factors of the 16 variables [17, 18]. These factors were rotated by the oblimin criterion [17]. A sample size of at least 200 patients offers adequate statistical power for factor analysis, especially since the ratio of the number of patients (n = 217) to the number of variables was also greater than 10 [19]. Last, to plot similarities between variables, a hierarchical cluster analysis was performed based on a similarity matrix that contains pairwise absolute Spearman correlation coefficients [17]. Analyses were performed using R, version 3.6.1 [20], psych package [21].

Reliability of continuous variables was evaluated using repeated measurements taken two months apart on 22 randomly selected subjects (10% of total). Bland–Altman plots, intraclass correlation coefficients (ICC), mean absolute differences and coefficients of variability (CV) [22] were calculated. To deal with negative values, the CV was calculated in three steps based on the recommendations of Martin Bland and Douglas Altman [23]. First, $s^2=\left(x-y\right)^2/2\;and\;m=\left(abs\left(x\right)+abs\left(y\right)\right)/2$ of a pair (x, y) were calculated; second, *s*^2^*m*^2^ =* of s*^2^/*m*^2^ was calculated; finally, the within-subject CV was the square root of mean(s^2^m^2^), which was expressed as percentage (*100). For the single examiner, the ICC(2,1) was calculated [24].

Ethical approval for the retrospective evaluation of archived, pseudonymized X-rays was granted from the Scientific Ethical Committee of Greifswald University Medicine (Reg.-No. BB134/15).

## Results

Regardless of the type of cephalometric analysis used, fundamentally comparable aspects of craniofacial morphology were examined, for which equivalent results were obtained. The cleft patients studied showed a retroposition of the maxilla and mandible, as well as the tendency towards an increased lower facial height and enlarged cranial base angle. In addition, the Delaire analysis examined aspects of craniofacial base relation and craniospinal articulation. Table 4 presents the patient characteristics; additional data (skewness, kurtosis) are given in Online Resource 1. A synopsis of related and unpaired variables can be found in Table 5.

**Table 4:**
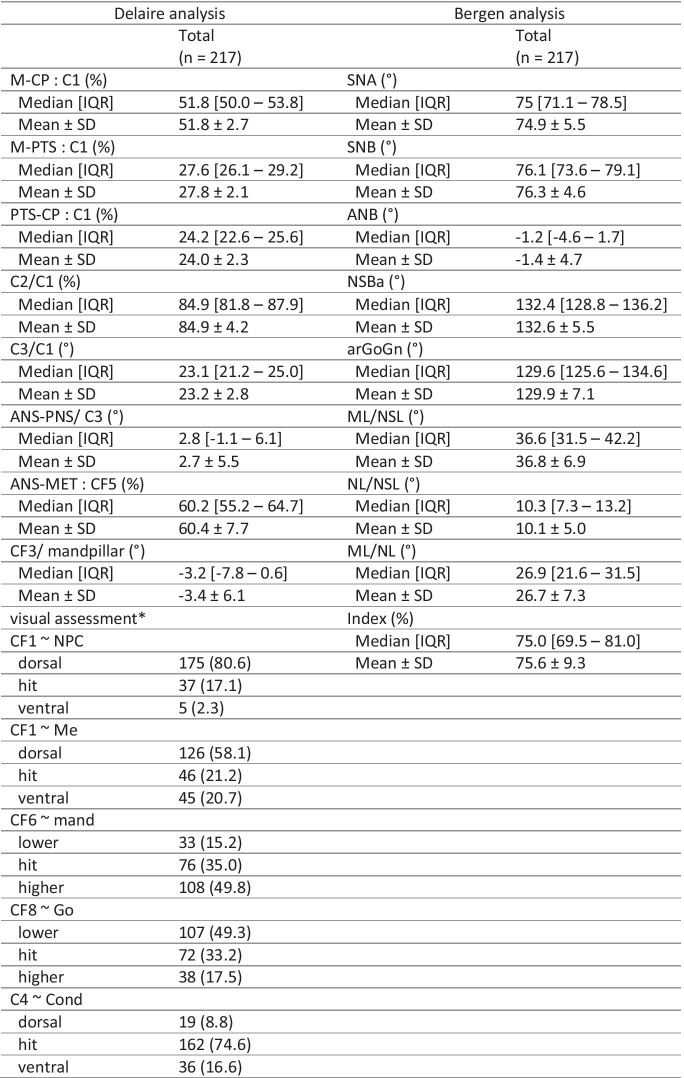
Patient characteristics

Values are number of patients (%) unless stated otherwise

^*^reference line passes through reference point (= hit), resp. dorsal/ventral or lower/higher

**Table 5:**
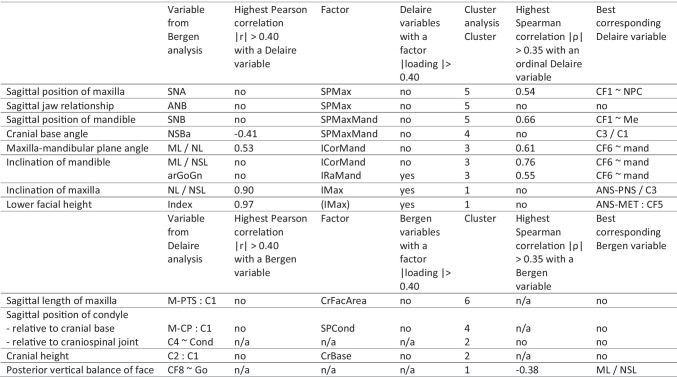
Synopsis of related and unpaired variables

### Correlation analysis

**Fig. 3:**
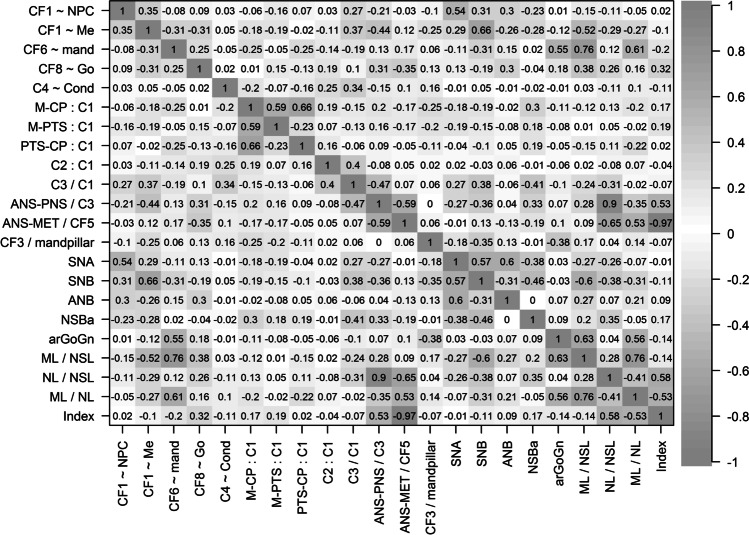
Correlation analysis of variables from two different approaches (Delaire, Bergen). For the first five variables, Spearman correlation coefficients are given; otherwise Pearson correlation coefficients are presented. The different shades of gray emphasize the absolute correlation, ignoring the sign

Some variables of the Delaire analysis were substantially correlated with those of the Bergen analysis (Fig. 3). CF1 ~ NPC showed the highest correlation with SNA (0.54); both variables evaluate the sagittal position of the maxilla. CF1 ~ Me was highly correlated with SNB (0.66); both variables describe the sagittal position of the mandible. The negative correlation with ML/NSL (− 0.52), mandibular inclination, can be explained by mutual dependency. CF6 ~ mand was correlated with arGoGn (0.55), ML/NSL (0.76) and ML/NL (0.61); all of them evaluate the mandibular inclination. C3/C1 showed some correlation with NSBa (0.41); both quantitate the cranial base angle. ANS-PNS/C3 was highly correlated with NL/NSL (0.90); both variables evaluate the inclination of the maxilla relative to the cranial base. The correlation with Index (0.53) can be explained by mutual dependency. ANS-MET:CF5 was highly correlated with Index (0.97); both quantitate the lower facial height. Owing to mutual dependency, ANS-MET:CF5 was also correlated with the variables of maxillary inclination NL/NSL (− 0.65) and mandibular inclination ML/NL (0.53). The other variables of the Delaire analysis showed no considerable correlations with variables of the Bergen analysis.

### Factor analysis

**Fig. 4:**
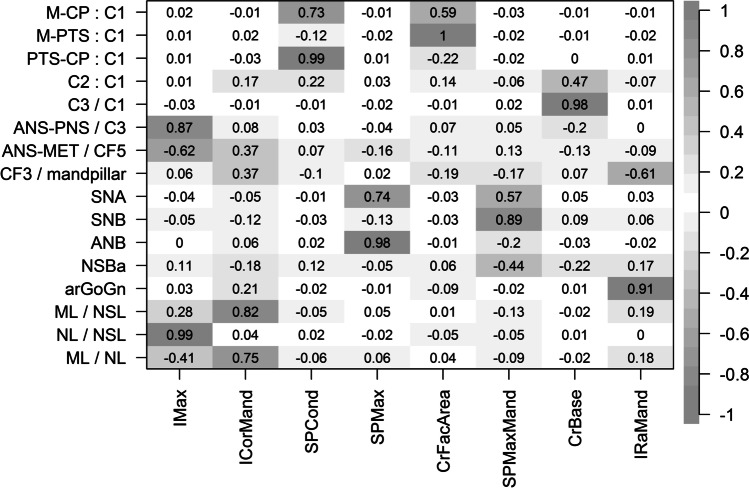
Factor analysis of continuous variables from two different approaches (Delaire, Bergen). The eight factors are denoted by Imax (inclination of maxilla), ICorMand (inclination of corpus mandibulae), SPCond (sagittal position of condyle), SPMax (sagittal position of maxilla), CrFacArea (relative length of craniofacial area), SPMaxMand (sagittal position of maxilla and mandible), CrBase (cranial base configuration) and IRaMand (inclination of ramus mandibulae). The different shades of gray emphasize the absolute correlation, ignoring the sign

The factor analysis reduced the 16 variables to eight factors (Fig. 4). Some factors were composed by high loadings of variables from both the Delaire analysis and the Bergen analysis (IMax, IRaMand). Other factors showed high loadings of variables attributed to either the Bergen analysis (ICorMand, SPMax, SPMaxMand) or the Delaire analysis (SPCond, CrFacArea, CrBase). This indicates that information about the inclination of the maxilla (IMax) and the inclination of the ramus mandibulae (IRaMand) can be obtained from continuous variables of both analyses. The inclination of corpus mandibulae (ICorMand), the sagittal position of the maxilla (SPMax) and the sagittal position of maxilla and mandible relative to the cranial base (SPMaxMand) were only evaluated by continuous variables of the Bergen analysis. Sagittal position of the condyle (SPCond), the relative length of the craniofacial area (CrFacArea) and the configuration of the cranial base (CrBase) were only assessed by the Delaire analysis.

### Cluster analysis

**Fig. 5:**
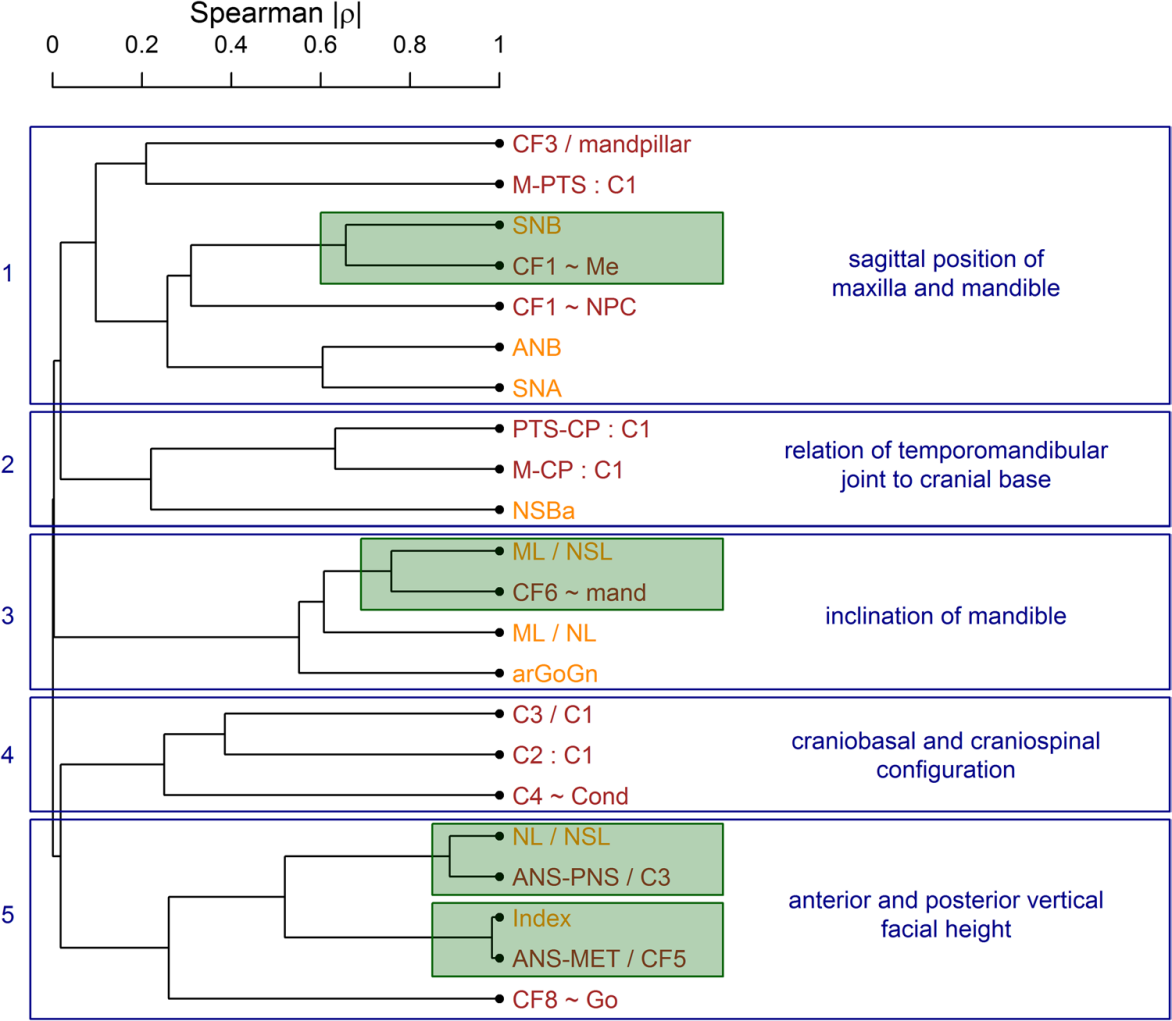
Hierarchical cluster analysis from two different approaches (Bergen, Delaire). The dendrogram shows which variables are similar in terms of the absolute Spearman correlation coefficient. Variables of the Bergen analysis are printed in orange; variables of the Delaire analysis are printed in dark brown. Particularly close clusters of variables from the Bergen and the Delaire analysis are highlighted in green

The dendrogram displays five main clusters (Fig. 5). Cluster 1 includes variables from both the Delaire and the Bergen analyses that describe the sagittal position of the maxilla and mandible. Note the close clustering of SNB and CF1 ~ Me. Cluster 2 describes the relation of the temporomandibular joint to the cranial base by similarities of two variables of the Delaire analysis (PTS-CP:C1 and M-CP:C1) and one of Bergen analysis (NSBa). Cluster 3 contains variables that describe the inclination of the mandible. Note the close cluster of ML/NSL and CF6 ~ Me. Cluster 4 only includes variables of the Delaire analysis (C3/C1, C2:C1 and C4 ~ Cond), all of which evaluate the general craniobasal and craniospinal configuration. Cluster 5 describes the anterior and posterior vertical facial height. Note the very close clusters of the variables of maxillary inclination (NL/NSL and ANS-PNS/C3) and of anterior facial height (Index and ANS-MET:CF5).

### Interrelationships among multivariate analyses

Based on the factor analysis, some aspects seem to be evaluated by the Bergen analysis only. However, taking into account the results of the cluster analysis, links to ordinal variables from the Delaire visual analysis can be found. Factor ICorMand correlated with the ordinal variable CF6 ~ mand (cluster 3). Factors SPMax and SPMaxMand correlated with the ordinal variables CF1 ~ Me and CF1 ~ NPC (cluster 1). Complemented by the visual evaluation, the Delaire analysis thus covers all aspects of the Bergen analysis. Regarding the factors that show high loadings of the Delaire variables only, some links to the Bergen analysis can be found. Factor SPCond correlated with variable NSBa (cluster 2). The Bergen analysis, though, does not provide direct information about the relative length of the maxillofacial field (M-PTS:C1) or mandibulofacial field (PTS-CP:C1). Correlations of factor CrFacArea to cluster 1 and cluster 2 are based on mutual dependency. Factor CrBase links to cluster 4. The variable of cranial base inclination, C3/C1, showed some correlation to NSBa. Other than that, no link to comparable variables from the Bergen analysis were found in any of the multivariate analyses conducted, indicating that the aspects of relative cranial height (C2:C1) and craniospinal articulation (C4 ~ Cond) are only assessed by the Delaire analysis.

### Reliability of measurements

With the exception of ANS-PNS/C3, all variables showed very good reproducibility in the Bland–Altman plots; the number of observations outside the limits of agreement was not critical (Table 6). The 95% CIs of the ICCs were very good to excellent. For variables close to or around zero, including ANB, NL/NSL, ANS-PNS/C3, and CF3/mandpillar, the coefficient of variability as a measure of the relative magnitude of error was high as expected for numerical reasons alone.

**Table 6:**
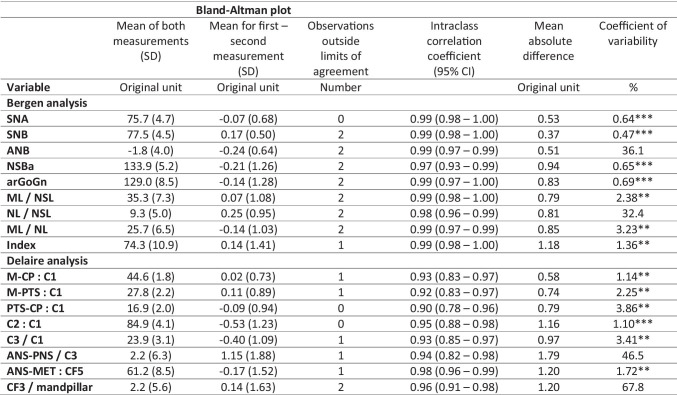
Measures of reliability

^*^ Good; ** very good; *** excellent

## Discussion

The findings suggest that most variables of the Delaire whole-skull analysis show similarities to variables of the Bergen analysis owing to comparable underlying results in the cephalometric evaluation of cleft patients. Thus, the inclusion of landmarks less compromised by the cleft defect or located outside the affected area does not appear to provide a viscerocranial assessment that differs from the one obtained by conventional landmarks. Certain aspects of the craniofacial base relation and the craniospinal articulation, however, are only assessed by the Delaire analysis.

### Common aspects of the Bergen analysis and the Delaire analysis

The sagittal position of the maxilla (point A) and mandible (point B) relative to the cranial base (S–N) is described by angles SNA and SNB in the Bergen analysis. Both angles show good reproducibility [25], but have been criticized for being influenced by the patient’s dentoalveolar frame, age and gender [3, 26], and for being subject to errors of interpretation in cleft patients [27–29]. The Delaire analysis employs visual assessment to evaluate the sagittal position of the maxilla and mandible (CF1 ~ NPC, CF1 ~ Me). In ideal conditions of craniofacial balance, CF1 passes through the anterior edge of the superior foramen of the nasopalatine canal (NPC) and through menton (Me). The accuracy of identification of landmark NPC depends on the quality of the radiograph [30]; difficulties in locating NPC and Me are considered a main weakness of the Delaire analysis [25]. Despite the objections raised for the variables of both the Bergen analysis and the Delaire analysis, we found similar results by means of correlation analysis and cluster analysis, suggesting that the variables serve the same purpose. This is particularly interesting in view of the results of Han et al., who have found the clinical paranasal diagnosis to be statistically significant correlated to the newly defined variable SN_NP_ (in the absence of upper lip procumbency or protrusion), but not to SNA [3]. The difference is possibly due to the fact that Han et al. performed a three-dimensional cephalometric analysis of the maxilla using cone-beam computed tomography scans of non-cleft patients, whereas we used two-dimensional cephalometric X-rays of cleft patients.

The variables of maxillary inclination — NL/NSL and ANS-PNS/C3 — were closely related in all three statistical analyses. Although both variables use landmarks that are prone to measurement error [28] and their relative measurement errors (in terms of the CV) were high, it can be assumed that these variables share important properties: First, the reliability in terms of the ICC was very good. Second, the Spearman correlation used in the cluster analysis is a rank correlation and therefore robust to some measurement error. Finally, both variables use closely related landmarks to define the maxillary plane (NL = ANS-Pm). This also suggests that the differences in defining the cranial base (NSL = N-S resp. C3 = M-Clp) appear to have no major impact. The high correlation of both variables (0.90) may also stem from the fact that the landmarks defining the cranial base share an anatomical structure (N and M: nasofrontal suture, S and Clp: sphenoid bone): According to Solow [31] correlations between variables can be expected if they involve a common reference structure.

In terms of mandibular inclination, we found similarities between the variables ML/NSL, ML/NL and CF6 ~ mand by means of correlation analysis and cluster analysis. A tangent following the inferior mandibular border (ML and mand) is related to the cranial base (NSL) and maxillary plane (NL) in the Bergen analysis and to the squamous occipital bone (CF6) in the Delaire analysis. However, the inclusion of the occiput does not seem to have a major impact on the result of the evaluation, especially since the inclination of line CF6 is also determined by the viscerocranium due to its dependence on Me', which also links the variable to the reference lines NSL (Na’) and NL (ANS).

The inclination of the ramus mandibulae is evaluated by both the Bergen analysis and the Delaire analysis. However, while arGoGn relates the inclination to the mandibular plane (Go-Gn), CF3/mandpillar relates it to the posterior line of craniofacial balance (CF3). Due to the dependence on CF2 and thus the position of Bregma, line CF3 can be considered a whole-skull variable. Nevertheless, arGoGn and CF3/mandpillar evaluate the inclination of the ramus mandibulae in a comparable way, as suggested by means of factor analysis.

The variables of lower facial height showed a high correlation and clustering. In the Bergen analysis, lower facial height is assessed by the Index, defined as the ratio of upper face length (N-Sp’) to lower face length (Sp’-Gn). The Delaire analysis evaluates the proportional share of ANS-MET at the theoretical facial height (CF5). Interestingly, although Nasion (Na) has been criticized for its variability and instability [28], Na’ (the projection of Nasion) is used here as upper limit of CF5. In contrast to Index and due to the dependency on CF4 and thus on C3, the inclination of CF5 is also determined by the cranial base (Clp).

Furthermore, the variables determining the cranial base angle — NSBa and C3/C1 — seem to provide comparable information and show some correlation. Its occurrence in cluster 2 also links NSBa to the sagittal position of the condyle (factor SPCond). This finding can be explained by the assumption that the shape and size of the cranial base have influence on the anteroposterior position of the condyle [32].

The variable describing the balance of the mandibular angle in the Delaire analysis — CF8 ~ Go — shows some correlation (− 0.38) to ML/NSL and appears in cluster 1, as ML/NSL does. Both variables evaluate the inclination of the mandible. However, while ML/NSL relates the mandibular plane to the cranial base, CF8 ~ Go relates the midpoint of the mandibular angle to the junction of the frontomaxillary/nasofrontal/nasomaxillary suture (M), the articular tubercle (CT) and the anterior nasal spine (ANS).

Considering that we found many common aspects of the Delaire analysis and the Bergen analysis, both analyses generally seem to provide comparable information. This finding supports the results of Brevi et al. [5], who have found no significant difference between a conventional cephalometric analysis (Steiner analysis) and Delaire analysis in preoperative diagnoses of patients with obstructive sleep apnoea syndrome. However, due to the reliance on individual harmonic whole-skull proportions, the Delaire analysis may not give the same surgical objectives as conventional cephalometric analyses [5, 6]. Also, except those variables evaluating the mandible (CF6 ~ mand, C4 ~ Cond, CF3 ~ mandpillar), most Delaire variables that have corresponding Bergen variables are mainly based on viscerocranial landmarks. These landmarks can then be supplemented by cranial points to assess the whole skull.

### Aspects solely described by the Delaire analysis

The length of the maxillofacial field (M-PTS:C1) and the sagittal position of the condyle relative to the cranial base and to the craniospinal joint (M-CP:C1 and C4 ~ Cond) are only evaluated by the Delaire analysis. As part of the whole-skull evaluation, the Delaire analysis also assesses cranial height (C2:C1). The Bergen analysis does not include corresponding variables.

The whole-skull information obtained through the Delaire analysis provide an overall view of the harmony and disharmony of the craniofacial complex. This can be beneficial for targeted research questions or when planning and assessing surgical advancement procedures. The finding is in line with Lippold et al. who have described a greater informative value of the Delaire analysis compared with a standard cephalometric analysis for assessing the individual cranial structural changes caused by LeFort III-distraction osteogenesis [4].

### Problems and revised concepts of the analyses

For reasons of radiation protection, many modern cephalograms do not depict the whole neurocranium and are therefore not suitable for the implementation of a whole-skull analysis such as the Delaire analysis. Various suggestions have been made in the literature on how to integrate the Delaire analysis into modern diagnostics, such as using the uncollimated areas in Digital Luminescence Radiography [33, 34], reconstructing lateral cephalograms from computed tomography (CT) -scans [35] or transitioning Delaire’s concept into a three-dimensional version based on CT-scans [36].

Contrary to the Delaire analysis, the classic Bergen analysis relies on normative values based on population means and standard deviations. Such values have been subject to criticism [37]. Therefore, in order to analyze skeletal patterns on an individualized basis, Segner has introduced “floating norms” for the Bergen analysis that derive from the patient’s individual facial type [38]. This supplement has nowadays become standard in clinical implementation of the Bergen analysis.

From a statistical point of view, non-numerical results like those deriving from Delaire’s concept of visual assessment can cause problems by categorization [39]. In a revised version of the analysis, Delaire has proposed the construction of additional auxiliary lines whose deviation from the reference line can be measured numerically [15].

## Conclusion

The Delaire analysis offers a comprehensive visualization of the patient’s individual sagittal and vertical craniofacial proportions and takes into account aspects that go beyond the scope of the Bergen analysis (for example, cranial height). From an orthodontic standpoint and within the scope of its viscerocranial field of view, though, the Bergen analysis appears to be on a par with the Delaire analysis and both are suitable for the cephalometric evaluation of cleft patients.

## Supplementary Information

Below is the link to the electronic supplementary material.

Online Resource 1 Patient characteristics (including skewness and kurtosis) (PDF 119 KB)

## Data Availability

The data underlying this article are available in Mendeley Data at http://dx.doi.org/10.17632/p9bpv7mzn3.1.
