# Supplementary material for: Cephalometric analyses for cleft patients: a statistical approach to compare the variables of Delaire’s craniofacial analysis to Bergen analysis
Source: Clin Oral Investig. 2021 May 29;26(1):353–64. doi: 10.1007/s00784-021-04006-3 (PMC8791903; doi:10.1007/s00784-021-04006-3)
Supplement: Supplementary file 1 — Patient characteristics (including skewness and kurtosis) (PDF 119 KB) [file 784_2021_4006_MOESM1_ESM.pdf]

Cephalometric analyses for cleft patients: A statistical approach to compare the variables of Delaire's craniofacial analysis to Bergen analysis

Clinical Oral Investigations

Philine H. Doberschütz*, Christian Schwahn, Karl-Friedrich Krey

*Department of Orthodontics, University Medicine Greifswald, Greifswald, Germany; philine.doberschuetz@uni-greifswald.de

Delaire analysis		Bergen analysis	
	Total (n = 217)		Total (n = 217)
M-CP : C1 (%)		SNA (°)	
Median [IQR]	51.8 [50.0 – 53.8]	Median [IQR]	75.0 [71.1 – 78.5]
Mean ± SD	51.8 ± 2.7	Mean ± SD	74.9 ± 5.5
Skewness; kurtosis*	-0.15; 2.71	Skewness; kurtosis	0.06; 2.84
M-PTS : C1 (%)		SNB (°)	
Median [IQR]	27.6 [26.1 – 29.2]	Median [IQR]	76.1 [73.6 – 79.1]
Mean ± SD	27.8 ± 2.1	Mean ± SD	76.3 ± 4.6
Skewness; kurtosis	0.56; 3.17	Skewness; kurtosis	0.14; 2.75
PTS-CP : C1 (%)		ANB (°)	
Median [IQR]	24.2 [22.6 – 25.6]	Median [IQR]	-1.2 [-4.6 – 1.7]
Mean ± SD	24.0 ± 2.3	Mean ± SD	-1.4 ± 4.7
Skewness; kurtosis	-0.50; 3.99	Skewness; kurtosis	-0.25; 3.14
C2/C1 (%)		NSBa (°)	
Median [IQR]	84.9 [81.8 – 87.9]	Median [IQR]	132.4 [128.8 – 136.2]
Mean ± SD	84.9 ± 4.2	Mean ± SD	132.6 ± 5.5
Skewness; kurtosis	0.25; 2.67	Skewness; kurtosis	0.25; 2.69
C3/C1 (°)		arGoGn (°)	
Median [IQR]	23.1 [21.2 – 25.0]	Median [IQR]	129.6 [125.6 – 134.6]
Mean ± SD	23.2 ± 2.8	Mean ± SD	129.9 ± 7.1
Skewness; kurtosis	0.36; 3.66	Skewness; kurtosis	0.22; 3.76
ANS-PNS/ C3 (°)		ML/NSL (°)	
Median [IQR]	2.8 [-1.1 – 6.1]	Median [IQR]	36.6 [31.5 – 42.2]
Mean ± SD	2.7 ± 5.5	Mean ± SD	36.8 ± 6.9
Skewness; kurtosis	0.07; 3.01	Skewness; kurtosis	0.16; 2.81
ANS-MET : CF5 (%)		NL/NSL (°)	
Median [IQR]	60.2 [55.2 – 64.7]	Median [IQR]	10.3 [7.3 – 13.2]
Mean ± SD	60.4 ± 7.7	Mean ± SD	10.1 ± 5.0
Skewness; kurtosis	0.26; 3.06	Skewness; kurtosis	-0.06; 3.33
CF3/ mandpillar (°)		ML/NL (°)	
Median [IQR]	-3.2 [-7.8 – 0.6]	Median [IQR]	26.9 [21.6 – 31.5]
Mean ± SD	-3.4 ± 6.1	Mean ± SD	26.7 ± 7.3
Skewness; kurtosis	0.001; 2.64	Skewness; kurtosis	0.11; 2.52
visual assessment**		Index (%)	
CF1 ~ NPC		Median [IQR]	75.0 [69.5 – 81.0]
dorsal	175 (80.6)	Mean ± SD	75.6 ± 9.3
hit	37 (17.1)	Skewness; kurtosis	0.32; 3.09
ventral	5 (2.3)		
CF1 ~ Me			
dorsal	126 (58.1)		
hit	46 (21.2)		

ventral	45 (20.7)
CF6 ~ mand	
lower	33 (15.2)
hit	76 (35.0)
higher	108 (49.8)
CF8 ~ Go	
lower	107 (49.3)
hit	72 (33.2)
higher	38 (17.5)
C4 ~ Cond	
dorsal	19 (8.8)
hit	162 (74.6)
ventral	36 (16.6)

Values are number of patients (%) unless stated otherwise
*a normal distribution would have a skewness of 0 and a kurtosis of 3
**reference line passes through reference point (=hit), resp. dorsal/ventral or lower/higher
